# Probiotic Supplement Use and Diabetic Retinopathy in Korean Adults with Diabetes: A Nationwide Population-Based Study

**DOI:** 10.3390/nu18213501

**Published:** 2026-10-25

**Authors:** Doh Hoon Chung, Jiwon Baek, Sung Pyo Park, Sean Hyungwoo Kim, Donghyun Jee

**Affiliations:** 1 Department of Ophthalmology and Visual Science, St. Vincent’s Hospital, College of Medicine, The Catholic University of Korea, Suwon 16247, Republic of Korea; 2 Apgujeong St. Mary’s Eye Center, 859 Eonju-ro, Gangnam-gu, Seoul 06023, Republic of Korea; 3 Department of Ophthalmology, Kangdong Sacred Heart Hospital, Hallym University College of Medicine, Seoul 05355, Republic of Korea; 4 Department of Biopharmaceutics and Pharmacogenomics, Bernard J. Dunn School of Pharmacy, Shenandoah University, Winchester, VA 22601, USA; skim3@su.edu

**Keywords:** diabetic retinopathy, probiotics, dietary supplements, gut–retina axis, gut microbiome, vision-threatening diabetic retinopathy, KNHANES, cross-sectional study

## Abstract

**Background/Objectives:** Experimental studies implicate the gut microbiome in diabetic retinopathy (DR), but population-based data on probiotic supplementation and DR are lacking. We examined this association in Korean adults with diabetes. **Methods:** We analyzed 2071 adults with diabetes in the Korea National Health and Nutrition Examination Survey 2018–2021 with graded fundus photographs and a product-level dietary supplement interview. We used survey-weighted logistic regression (prespecified) and Poisson regression (added after the initial analysis) estimated odds ratios (ORs) and prevalence ratios (PRs), adjusted for age, sex, hypertension, smoking, and household income (Model 1), and additionally for diabetes duration and glycated hemoglobin (HbA1c) (Model 2, primary), with further adjustment for clinical, lifestyle, and dietary factors. **Results:** Probiotic supplements were used by 10.4% of participants. Weighted DR prevalence was 11.0% among users and 21.1% among non-users (Model 2 PR 0.57, 95% CI 0.36–0.90; OR 0.49, 95% CI 0.28–0.86; 27 exposed cases; fully adjusted PR 0.59, 95% CI 0.37–0.95). In exploratory analyses, compared with non-use, the PRs were 0.76 (95% CI 0.45–1.29; not statistically significant) for use of <1 year and 0.28 (95% CI 0.13–0.62) for use of ≥1 year. No probiotic user had vision-threatening DR, compared with 3.9% of non-users (unadjusted design-based *p* = 0.024), a descriptive observation that does not demonstrate a protective effect. Use of any non-probiotic supplement was not associated with DR (PR 0.92), although vitamin C and vitamin A/lutein supplements showed inverse associations of similar magnitude. **Conclusions:** Probiotic supplement use was associated with a lower prevalence of DR among Korean adults with diabetes. These cross-sectional findings are consistent with, but do not establish, a role of the gut–retina axis in DR and require confirmation in prospective studies.

## 1. Introduction

Diabetic retinopathy (DR) remains the leading cause of preventable blindness among working-age adults. Approximately 103 million people were living with DR in 2020, and this number is projected to reach 160 million by 2045, with a disproportionate rise in Asia [1,2]. In Korea, DR affects 11–16% of adults with diabetes, and vision-threatening DR (VTDR) about 5%, with diabetes duration and glycated hemoglobin (HbA1c) being the dominant risk factors [3,4]. Because intensive glycemic control only partly prevents retinopathy and current treatments target late, sight-threatening stages, modifiable factors that act on the earlier microvascular and inflammatory phases of the disease are of considerable public health interest.

The gut microbiome has emerged as such a factor. The concept of a gut–retina axis was introduced when a low-glycemic diet was shown to arrest age-related macular degeneration (AMD)-like retinal changes in mice through a shift in gut microbial composition and metabolites [5], and rodent and human studies have since extended this axis to DR [6,7]. People with type 2 diabetes and DR harbor a distinct gut microbiome characterized by loss of anti-inflammatory and probiotic taxa, reduced microbial diversity, and an altered Bacteroidetes to Firmicutes ratio, and dysbiosis is more pronounced in DR than in diabetes without retinopathy [8,9,10,11]. In db/db mice, restructuring of the gut microbiome by intermittent fasting prevented retinal capillary loss without changing HbA1c, an effect attributed to microbial bile-acid metabolites acting on retinal TGR5 receptors [12]. In type 1 diabetic humans, markers of gut barrier leakage correlate with DR severity, and oral administration of Lactobacillus strains genetically engineered to express human angiotensin-converting enzyme 2 (ACE2) or angiotensin-(1–7) to diabetic mice preserved the gut barrier, lowered systemic inflammation, and prevented or reversed retinopathy without improving glycemia [13,14,15,16]. These experimental findings suggest that orally delivered probiotic bacteria could modulate the gut–retina axis, although this has not been tested in humans.

In humans, randomized trials show that probiotic supplementation modestly improves fasting glucose, insulin resistance, lipids, blood pressure, and inflammatory markers in type 2 diabetes [17,18], and improves metabolic and oxidative stress profiles in diabetic patients with kidney disease [19]. No clinical trial, however, has examined DR as an outcome, and, to our knowledge, no population-based study has tested whether probiotic supplement use is related to the presence or severity of DR. Meanwhile, the use of probiotic supplements has grown rapidly; in Korea, probiotics are now among the most commonly consumed dietary supplements and probiotic foods [20,21]. The Korea National Health and Nutrition Examination Survey (KNHANES) offers a unique opportunity to address this question because it combines a product-level dietary supplement interview with standardized fundus photography graded by retinal specialists in a nationally representative sample.

We therefore investigated the association between probiotic supplement use and DR, including its severity, diabetic macular edema (DME), and VTDR, among Korean adults with diabetes in KNHANES 2018–2021, with exploratory subgroup analyses by sex and age. As exploratory analyses, we also examined other retinal diseases assessed in the survey, namely AMD, epiretinal membrane (ERM), and branch retinal vein occlusion (BRVO).

## 2. Materials and Methods

### 2.1. Study Population

KNHANES is an ongoing nationwide cross-sectional survey conducted by the Korea Disease Control and Prevention Agency (KDCA) that uses a stratified, multistage, clustered probability sampling design to select a representative sample of the non-institutionalized Korean population; it comprises a health interview, a health examination, and a nutrition survey [22]. We used data from the third year of the seventh survey (2018) and the entire eighth survey (2019–2021), the years in which the ophthalmic examination included fundus photography and the nutrition survey included the product-level dietary supplement questionnaire. The ophthalmic examination targeted participants aged 40 years or older in 2018–2020 and those aged 10–59 years in 2021, as described previously [23]. Consequently, no adult aged 60 years or older contributed ophthalmic data in 2021; because survey weighting cannot fully correct for this change in design, we repeated the analyses after excluding the 2021 data and after restricting the sample to adults aged 40 years or older. The survey protocol was approved by the Institutional Review Board of the KDCA, and all participants provided written informed consent; the present analysis of de-identified public data was exempted from review by the Institutional Review Board of St. Vincent’s Hospital, College of Medicine, the Catholic University of Korea (IRB No. VC17ZESI0207), and adhered to the tenets of the Declaration of Helsinki.

**Figure 1:**
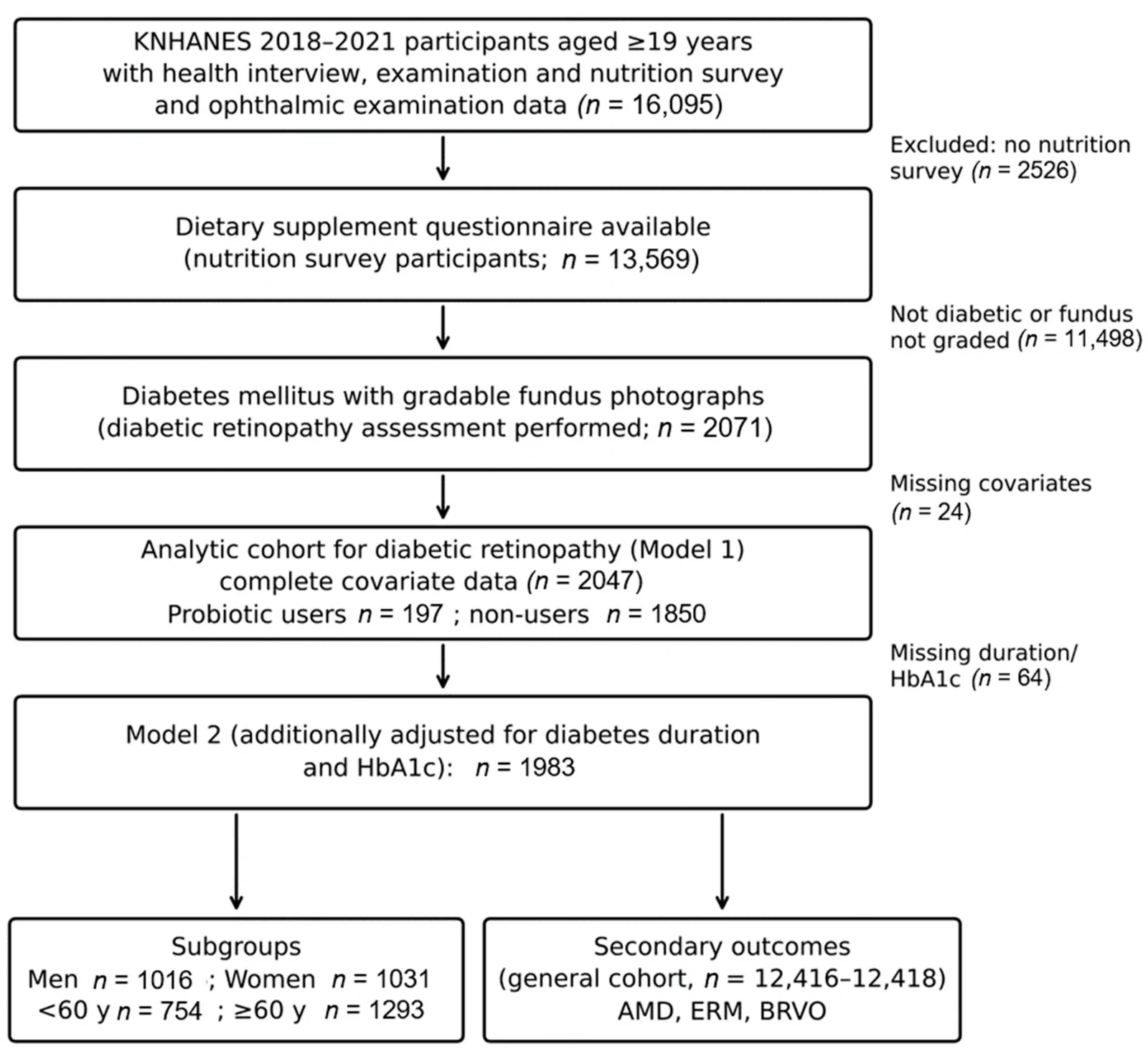
Flow of participants. KNHANES, Korea National Health and Nutrition Examination Survey; AMD, age-related macular degeneration; ERM, epiretinal membrane; and BRVO, branch retinal vein occlusion.

Of 16,095 participants aged 19 years or older with ophthalmic examination data, 13,569 completed the nutrition survey and had dietary supplement information. Adults with diabetes in whom DR was graded from fundus photographs (*n* = 2071) formed the primary analytic cohort; 2047 participants had complete data for the Model 1 covariates and 1983 for the Model 2 covariates (Figure 1). Diabetes was defined by the KDCA composite variable as a physician diagnosis, current use of glucose-lowering medication or insulin, fasting plasma glucose ≥ 126 mg/dL, or HbA1c ≥ 6.5%; in the 2018 survey, the composite variable did not include the HbA1c criterion. Among the 2071 participants, 2010 were classified as having diabetes by the composite variable. The remaining 61, all examined in 2018, had an HbA1c of 6.5–8.0% (median 6.6%) and were retained because fundus grading for DR had been performed. Because diabetes duration was derived only for participants classified by the composite variable, it was missing for these 61 participants, who were therefore not included in Model 2; a sensitivity analysis also excluded them from Model 1. Of the 2202 participants who met the composite diabetes definition and had dietary supplement data, 192 (8.7%) had no gradable DR assessment; compared with those who were graded, they were older (mean 70.8 vs. 63.3 years), had a longer diabetes duration and a lower estimated glomerular filtration rate, and used probiotics less often (3.6% vs. 9.8%) (Table S3). The potential effect of this selection was examined with inverse probability weighting (Section 2.5).

### 2.2. Assessment of Probiotic Supplement Use

Dietary supplement use was ascertained by trained dietitians during the nutrition survey. Participants who reported current use of any dietary supplement were asked to show each product; for each product, the interviewer recorded the product name, manufacturer, functional category, duration of use (<1 month, 1–3, 4–6, 7–11 months, or ≥1 year), frequency of intake, and whether it was taken on the day before the survey. Products were classified by the KDCA into 12 categories: multivitamin-mineral, vitamin C, omega-3 fatty acids, probiotics, red ginseng, calcium, vitamin A/lutein, propolis, vitamin D, iron, other vitamins and minerals, and others [20]. Probiotic supplement use was defined as current use of one or more products in the probiotic category. Participants who used no dietary supplement or used only non-probiotic supplements were classified as non-users. For exploratory analyses of duration, probiotic users were categorized by the reported duration of use of their probiotic product as users of less than one year and users of one year or longer, with non-users as the common reference category.

### 2.3. Ophthalmic Examination and Outcome Definitions

The ophthalmic examination protocol of KNHANES 2017–2021 has been described in detail [23,24]. Digital non-mydriatic fundus photographs (45°) centered on the macula and optic disc were obtained from each eye by trained ophthalmologists using a digital fundus camera and were graded by retinal specialists of the Korean Ophthalmological Society epidemiologic survey committee according to standardized protocols; fundus photographs were additionally obtained after pupil dilation in participants with diabetes. DR was graded in participants with diabetes as none, mild to moderate non-proliferative DR (NPDR), severe NPDR, or proliferative DR (PDR) on the basis of the International Clinical Diabetic Retinopathy severity scale, and DME was recorded as present or absent [25]. Each participant was classified according to the worse eye. Any DR was the primary outcome. Mild to moderate NPDR was a secondary outcome; severe NPDR or PDR, PDR, DME, and VTDR, defined as severe NPDR, PDR, or DME, were exploratory outcomes. Eyes that could not be graded were excluded, and participants without a gradable eye had no DR assessment (Section 2.1). KNHANES does not record previous laser photocoagulation, intravitreal injection, or vitrectomy for DR; treated DR could therefore not be identified or excluded, and a sensitivity analysis excluded participants who reported any previous eye surgery. In the general cohort, AMD (early or late), ERM, and retinal vein occlusion were graded from fundus photographs; BRVO was defined as branch occlusion in either eye.

### 2.4. Covariates

Covariates for Models 1 and 2 were selected before the analysis on the basis of established risk factors for DR and determinants of supplement use; after the initial analysis, these assumptions were summarized in a directed acyclic graph (DAG; Figure S1), which was also used to select the additional covariates of Models 3 and 4. Model 1 included age, sex, hypertension, current smoking, and household income, which may influence both supplement use and DR. Model 2, the primary model, additionally included diabetes duration and HbA1c. Hypertension was defined according to the KDCA as systolic blood pressure ≥ 140 mmHg, diastolic blood pressure ≥ 90 mmHg, or use of antihypertensive medication. It was treated as a confounder because hypertension and its treatment usually precede the survey and can influence health behaviors, including supplement use, as well as the risk of DR; because this definition partly relies on blood pressure measured at the survey, which probiotics may modify [17,18], Model 2 was repeated with hypertension defined by antihypertensive medication use alone. Blood pressure, lipid, and fasting glucose levels measured at the survey were not included as continuous covariates, because they may lie partly on a pathway between probiotic use and DR. HbA1c may act both as a confounder and as a mediator; because a single cross-sectional measurement cannot separate these roles, Model 2 was regarded as a conservative model rather than as a test of mediation. Current smoking was self-reported. Household income was categorized into quartiles by the KDCA using equivalized household income. Diabetes duration was calculated as the difference between age at survey and self-reported age at physician diagnosis; participants classified as having diabetes by the composite variable without a physician diagnosis (that is, by screening glucose or HbA1c, or by medication use) were assigned a duration of 0 years. HbA1c was measured by high-performance liquid chromatography (Tosoh G8, Tokyo, Japan) in a central laboratory. Model 3 additionally included body mass index (BMI), calculated from measured height and weight; current insulin use; estimated glomerular filtration rate (eGFR), calculated with the 2021 CKD-EPI creatinine equation; education (four levels); area of residence (urban vs. rural); alcohol consumption at least once a month during the past year; and aerobic physical activity meeting the national guideline. Because BMI and kidney function may also be influenced by probiotics [19], Model 3 may be over-adjusted. Use of oral glucose-lowering drugs was recorded without the name of the agent, so metformin could not be examined separately; use of any oral glucose-lowering drug was examined in a sensitivity analysis. Model 4 additionally included fermented food intake (g/day), estimated from the 24 h dietary recall as the sum of kimchi, fermented soybean pastes, and fermented dairy products, and any consumption of fermented dairy products on the recall day.

### 2.5. Statistical Analysis

All analyses accounted for the complex survey design using the stratification, cluster, and sample weight variables provided by the KDCA. Because the eye examination, health examination, and nutrition survey were combined, the integrated survey weight (wt_tot) was used, and weights were rescaled for pooling across survey years in proportion to the survey period of each year (the eye examination was conducted for six months in 2019), as recommended in the KNHANES analytic guidelines [22]; the weights were multiplied by 1/3.5 for 2018, 2020, and 2021 and by 0.5/3.5 for 2019. Weighted prevalence estimates and means are reported with unweighted numbers. Differences in baseline characteristics between probiotic users and non-users were tested with design-based linear regression (Wald *t*-test).

Model 1, Model 2 (the primary model), the outcome definitions, and the odds ratio (OR) from survey-weighted logistic regression as the measure of association were specified before the analysis, but the analysis plan was not registered or published as a protocol. The primary analysis was the association between probiotic use and any DR in Model 2; Models 1, 3, and 4 and the analysis of mild to moderate NPDR were secondary analyses, and the analyses of DR severity, duration of use, subgroups, other supplements, and other retinal diseases, and all sensitivity analyses, were exploratory. All other analyses were specified after the initial analysis (post hoc), including the severity, subgroup, negative-control, and E-value analyses, the DAG, prevalence ratios, Models 3 and 4, interaction tests, design-based tests for severity outcomes, duration categories, all sensitivity analyses (a)–(o) below, the weight-distribution and effective-sample-size analyses, influence analyses, false discovery rate correction, and E-value benchmarking. Because DR was common (20%) and ORs therefore overstate the relative difference in prevalence, the prevalence ratio (PR) was chosen after the initial analysis as the main measure of association; it was estimated with survey-weighted Poisson regression with a log link [26] and Taylor-series linearization variance [27]; ORs from survey-weighted logistic regression, the measure specified before the analysis, are reported alongside the PRs. The choice of the PR did not change the prespecified primary model, outcome, or covariates. All analyses of the diabetic cohort, its subgroups, and complete-case samples were performed as domain (subpopulation) analyses of the full survey design, so that variances accounted for the random domain sample sizes [27]. Subgroup analyses were performed by sex and by age (<60 vs. ≥60 years), and heterogeneity was assessed with design-based Wald tests of product terms between probiotic use and sex or age group in Model 2 fitted to the whole cohort. For severity outcomes, no event occurred among probiotic users and model-based estimates were not calculated; the distribution of each outcome by probiotic use was compared with the design-based Rao–Scott chi-square test, without covariate adjustment, and unweighted Fisher exact tests are reported for descriptive purposes only. Duration of use was analyzed as a three-category variable (non-users, <1 year, and ≥1 year); a test for trend was obtained by entering the category as an ordinal term, and users of ≥1 year were also compared with users of <1 year within the same model.

Sensitivity analyses (a) restricted the reference group to participants who used no dietary supplement of any kind, (b) excluded the 61 participants who did not meet the composite diabetes definition, (c) excluded participants who reported previous eye surgery, (d) excluded insulin users, (e) restricted the sample to adults aged 40 years or older and (f) to the 2018–2020 surveys, (g) additionally adjusted Model 2 for survey year, (h) restricted the sample to participants with physician-diagnosed diabetes, (i) additionally adjusted Model 2 for use of oral glucose-lowering drugs and insulin, (j) defined hypertension by antihypertensive medication use alone, (k) applied stabilized inverse probability weights, multiplied by the survey weights, to Model 3 for having a gradable DR assessment (estimated among all participants with diabetes from age, sex, HbA1c, diabetes duration, eGFR, insulin use, hypertension, household income, probiotic use, area of residence, and survey year) and for having complete Model 3 covariates (estimated from age, sex, hypertension, probiotic use, DR, HbA1c, area of residence, and survey year), (l) truncated the survey weights at the 99th and 95th percentiles, (m) omitted the survey weights while retaining strata and clusters, (n) re-estimated Model 3 without the insulin-use term, with and without exclusion of insulin users, because only three probiotic users used insulin, and (o) performed a tipping-point analysis for participants with diabetes without a gradable DR assessment, because inverse probability weighting does not account for data missing not at random. In the tipping-point analysis, DR status was assigned to the 180 ungradable participants with complete Model 2 covariates (7 probiotic users) under scenarios in which the DR prevalence among ungradable non-users ranged from 10% to 60% and the number of ungradable probiotic users with DR ranged from 0 to 7, and Model 2 was re-estimated in the combined sample (*n* = 2163); each scenario was analyzed with 50 random assignments combined with Rubin’s rules. The influence of individual clusters was assessed by re-estimating Model 2 after omitting each primary sampling unit (PSU) in turn. To address confounding by health-conscious behavior (the healthy-user effect) [28], we performed negative-control exposure analyses [29] in which each of the 10 non-probiotic supplement categories with more than one user, and use of any non-probiotic supplement, was substituted for probiotic use in Model 2; if the probiotic association merely reflected a healthy-user effect, other supplements would be expected to show similar inverse associations. *p* values for the 11 individual supplement categories, including probiotics, were corrected with the Benjamini–Hochberg false discovery rate (FDR) method; use of any non-probiotic supplement, a composite of these categories, was not included. This correction applied only to the exploratory negative-control comparison, in which probiotics were included so that all categories were evaluated in the same way; the primary analysis (any DR, Model 2) was a single comparison specified before the analysis and was evaluated at the nominal significance level, so the FDR-adjusted values do not bear on its interpretation. The probiotic model was also mutually adjusted for use of any other supplement, for the number of other supplements, and for the multivitamin, vitamin C, omega-3, and vitamin A/lutein categories. E-values [30] were calculated from the Model 2 PR and compared with the associations of the measured covariates with DR and with probiotic use, estimated in mutually adjusted survey-weighted Poisson models (benchmarking). Exploratory outcomes in the general cohort (AMD, late AMD, ERM, and BRVO) were analyzed with Model 1 additionally adjusted for diabetes. A two-sided *p* < 0.05 was considered statistically significant for the primary analysis; other *p* values were not adjusted for multiple testing unless stated and are reported as nominal values. All analyses were performed with R 4.3.3 (survey package 4.5; survey package 4.2.1 for the analyses added in the second revision, which reproduced the primary estimates); initial estimates obtained with custom Python 3.10 code were verified in R.

## 3. Results

### 3.1. Characteristics of the Study Population

The diabetic cohort comprised 2071 adults (weighted mean age of 60.0 years; range of 22–80 years; 42.0% women). Probiotic supplements were currently used by 199 participants, corresponding to a weighted prevalence of 10.4%, which increased from 4.8% in 2018 to 9.5% in 2019 and 14.4% in 2020 (11.8% in 2021, when only adults younger than 60 years were examined). Among probiotic users, 86% took the product daily and 39% (77/199) had used it for at least one year. Compared with non-users, probiotic users were younger, more often women, less often current smokers, less often in the lowest income quartile, had slightly lower HbA1c, and a shorter, although not significantly different, diabetes duration. Probiotic users were also less often insulin users, had a higher eGFR, and were more often college educated and physically active, whereas BMI, area of residence, alcohol consumption, and fermented food intake did not differ (Table 1). The weighted prevalence of any DR was 20.0% in the whole diabetic cohort, 11.0% among probiotic users, and 21.1% among non-users.

### 3.2. Probiotic Use and Diabetic Retinopathy

**Figure 2:**
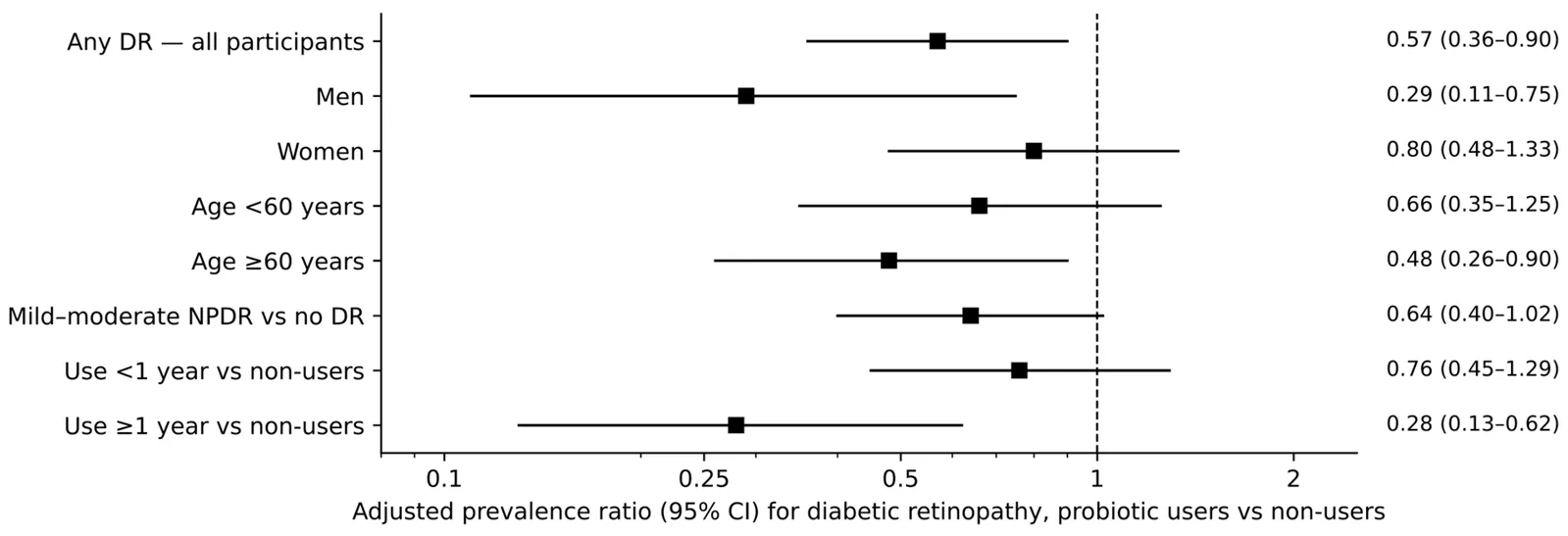
Adjusted prevalence ratios for diabetic retinopathy among probiotic supplement users compared with non-users. Prevalence ratios (95% confidence intervals) overall, by sex and age, for mild to moderate non-proliferative diabetic retinopathy versus no retinopathy, and by duration of probiotic use (<1 year and ≥1 year, each versus non-users). Estimates are from survey-weighted Poisson regression adjusted for age, sex, hypertension, current smoking, household income, diabetes duration, and HbA1c (Model 2); subgroup estimates are from domain analyses. Subgroup and duration analyses are exploratory. DR, diabetic retinopathy; NPDR, non-proliferative diabetic retinopathy.

Probiotic supplement use was associated with a lower prevalence of any DR in crude analysis (PR 0.52, 95% CI 0.33–0.82; OR 0.46, 95% CI 0.28–0.78) and after adjustment for age, sex, hypertension, smoking, and income (Model 1 PR 0.51, 95% CI 0.32–0.81; *p* = 0.004; OR 0.45, 95% CI 0.27–0.76). In the primary model, which additionally included diabetes duration and HbA1c, the PR was 0.57 (95% CI 0.36–0.90; *p* = 0.017) and the OR 0.49 (95% CI 0.28–0.86; *p* = 0.012), based on 27 DR cases among 195 exposed participants (Table 2, Figure 2). Further adjustment for BMI, insulin use, eGFR, education, residence, alcohol consumption, and physical activity (Model 3), and for fermented food intake (Model 4), changed the estimate little (both PR 0.59, 95% CI 0.37–0.95), and Model 2 gave the same estimate in these participants. The point estimate for mild to moderate NPDR was similar, but its confidence interval included unity (Model 2 PR 0.64, 95% CI 0.40–1.02; *p* = 0.059; OR 0.57, 95% CI 0.33–1.00; *p* = 0.049).

### 3.3. Diabetic Retinopathy Severity, Diabetic Macular Edema and Vision-Threatening Retinopathy

**Figure 3:**
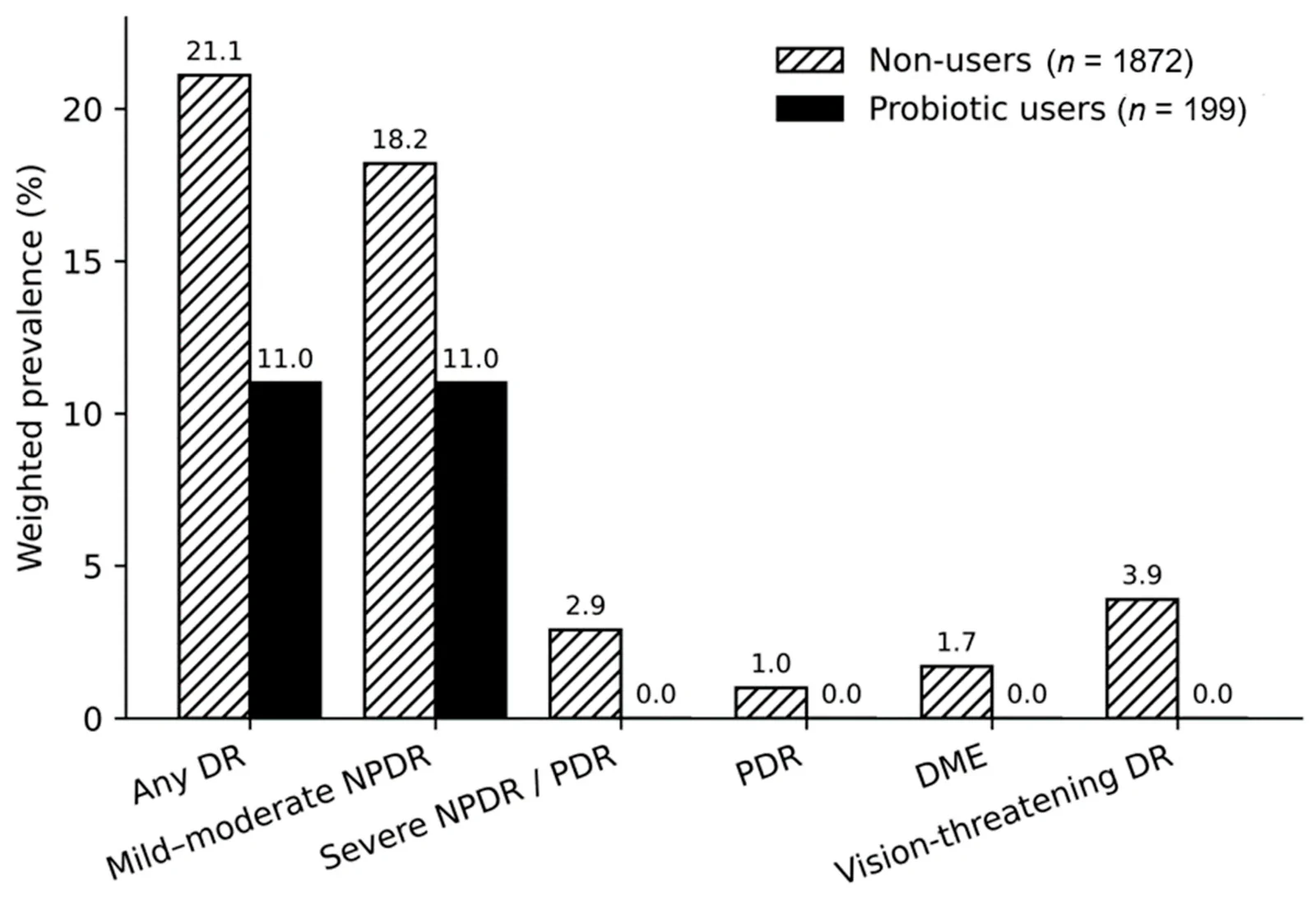
Prevalence of diabetic retinopathy by severity according to probiotic supplement use. Survey-weighted prevalence of diabetic retinopathy by severity, diabetic macular edema, and vision-threatening diabetic retinopathy among Korean adults with diabetes according to probiotic supplement use (KNHANES 2018–2021). No probiotic user had severe non-proliferative retinopathy, proliferative retinopathy, macular edema, or vision-threatening retinopathy. DR, diabetic retinopathy; NPDR, non-proliferative DR; PDR, proliferative DR; and DME, diabetic macular edema.

None of the 199 probiotic users had severe NPDR, PDR, DME, or VTDR, whereas among 1872 non-users the weighted prevalence of severe NPDR or PDR was 2.9% (58 participants), that of PDR 1.0% (27), of DME 1.7% (30), and of VTDR 3.9% (72) (Table 2, Figure 3). The exact 95% CI for the proportion of probiotic users with VTDR was 0–1.8%. With design-based Rao–Scott tests, the nominal *p* values were 0.024 for VTDR, 0.055 for severe NPDR or PDR, 0.17 for PDR, and 0.12 for DME; the corresponding unweighted Fisher exact *p* values were 0.0009, 0.005, 0.10, and 0.11. These tests do not account for differences in age, smoking, income, or other covariates between users and non-users, and because no event occurred in the exposed group, adjusted estimates could not be obtained; these results are therefore descriptive, refer to the observed sample only, and do not demonstrate a protective effect.

No probiotic user had VTDR in any stratum (Table S1), but the number of exposed participants in each stratum was small. The Fisher exact *p* value was 0.010 in women and 0.046 in adults aged 60 years or older, but 0.16 in men, 0.063 in adults younger than 60 years, and 0.25–0.60 in the individual survey years; compared with non-users, it was 0.020 for users of less than one year and 0.11 for users of one year or longer. These stratum-specific results are descriptive.

### 3.4. Subgroup Analyses

In exploratory domain analyses, the Model 2 PR for any DR was 0.29 (95% CI 0.11–0.75) in men and 0.80 (95% CI 0.48–1.33) in women (*p* for interaction = 0.058), and 0.66 (95% CI 0.35–1.25) in adults younger than 60 years and 0.48 (95% CI 0.26–0.90) in adults aged 60 years or older (*p* for interaction = 0.67) (Table 3, Figure 2); the corresponding interaction *p* values on the OR scale were 0.045 and 0.64. The estimates were below unity in every subgroup and their confidence intervals overlapped, and the estimate in men was based on seven exposed cases; given the number of comparisons and the absence of correction for multiple testing, these results do not establish effect modification. Weighted DR prevalence among probiotic users versus non-users was 6.6% versus 20.7% in men, 14.0% versus 21.6% in women, 11.5% versus 21.1% in adults younger than 60 years, and 10.2% versus 21.1% in adults 60 years or older.

### 3.5. Duration of Use and Sensitivity Analyses

Compared with non-users, the PR for any DR was 0.76 (95% CI 0.45–1.29) among users of less than one year (119 users, 20 cases) and 0.28 (95% CI 0.13–0.62) among users of one year or longer (76 users, 7 cases) (*p* for trend = 0.003; weighted DR prevalence 21.1%, 13.4% and 6.6%, respectively) (Table 4). The PR comparing use of one year or longer with use of less than one year was 0.37 (95% CI 0.15–0.91; *p* = 0.031). Because duration was self-reported, the number of long-term users was small, current long-term users are a selected group of persistent users, and the analysis was exploratory, this pattern should not be interpreted as a dose–response relationship. The association was similar when the reference group was restricted to participants who used no dietary supplement of any kind (PR 0.58, 95% CI 0.36–0.94), after exclusion of participants with previous eye surgery (PR 0.61, 95% CI 0.36–1.01) or of insulin users (PR 0.57, 95% CI 0.36–0.92), after restriction to adults aged 40 years or older (PR 0.57, 95% CI 0.36–0.90) or to the 2018–2020 surveys (PR 0.54, 95% CI 0.32–0.89), after additional adjustment for survey year (PR 0.56, 95% CI 0.35–0.90) or for use of oral glucose-lowering drugs and insulin (PR 0.60, 95% CI 0.38–0.96), with hypertension defined by antihypertensive medication use alone (PR 0.57, 95% CI 0.36–0.90), after restriction to participants with physician-diagnosed diabetes (PR 0.50, 95% CI 0.30–0.83), with inverse probability weighting for gradability and missing covariates (PR 0.60, 95% CI 0.37–0.95; stabilized weights 0.83–2.06, mean 0.99), and after truncation of the survey weights at the 99th or 95th percentile (PR 0.57 and 0.58, respectively). Excluding the 61 participants who did not meet the composite diabetes definition did not change the Model 1 estimate (PR 0.50, 95% CI 0.32–0.80). Only three insulin users took probiotics, so the association could not be examined among insulin users. Because of this small number, Model 3 was re-estimated without the insulin-use term (PR 0.58, 95% CI 0.36–0.93) and, in addition, after exclusion of insulin users (PR 0.58, 95% CI 0.36–0.95; *n* = 1779); the estimates were similar to that of Model 3 (Table 4). Omitting, one at a time, each of the 158 PSUs that contained probiotic users yielded PRs between 0.51 and 0.59. In the tipping-point analysis (Table S5), the confidence interval of the Model 2 PR included unity only when DR was assigned to at least three of the seven ungradable probiotic users (43%) with a DR prevalence of 10% among ungradable non-users, to at least four (57%) with a prevalence of 20% or 30%, to at least five (71%) with 40%, to at least six (86%) with 50% and to all seven with 60% (at six of seven, the upper limit was 0.999). When the DR prevalence assigned to ungradable probiotic users was closest to that assigned to ungradable non-users, the PR ranged from 0.58 to 0.63, and all confidence intervals excluded unity. The association would therefore be explained by the missing DR assessments only if DR was considerably more common among ungradable probiotic users than among ungradable non-users.

Without survey weights (retaining strata and clusters), the estimates were attenuated (Model 1 PR 0.67, 95% CI 0.46–0.98; Model 2 PR 0.73, 95% CI 0.50–1.07; OR 0.67, 95% CI 0.42–1.07). The Kish effective sample size of the 2071 participants was 1318 overall and 135 among probiotic users. Mean weights were similar in users and non-users (1923 vs. 1765; coefficient of variation 0.69 vs. 0.76; 1st, 50th, and 99th percentiles 237, 1585, and 5970 vs. 209, 1369, and 6778, respectively), but among probiotic users, those with DR had smaller weights than those without DR (mean 1499 vs. 1993), whereas this was not the case among non-users (1835 vs. 1747). Probiotic users with DR therefore represent fewer people in the population, which explains why the unweighted DR prevalence among users (14.1%) was higher than the weighted prevalence (11.0%), whereas that among non-users was similar (20.2% vs. 21.1%). The weights are designed to correct for unequal selection probabilities and non-response, and the weighted estimate was stable under weight truncation and PSU deletion. However, the difference between the weighted and unweighted estimates indicates that the weights are informative for this association, and the weighted estimate depends on the validity of the KDCA weights.

### 3.6. Negative-Control Exposures and E-Values

Use of any non-probiotic supplement, reported by 1003 participants with diabetes, was not associated with DR (Model 2 PR 0.92, 95% CI 0.75–1.14), and neither were multivitamin-mineral (PR 0.94), omega-3 fatty acid (PR 1.10), red ginseng (PR 0.95), calcium (PR 0.96), other vitamin and mineral (PR 0.81), or other supplements (PR 1.06) (Table 5). However, vitamin C (PR 0.62, 95% CI 0.39–1.00) and vitamin A/lutein (PR 0.58, 95% CI 0.35–0.98) supplements showed inverse associations of a magnitude similar to that of probiotics, vitamin D was not significantly associated with DR (PR 0.46, 95% CI 0.17–1.20), and propolis, used by only 34 participants, was positively associated (PR 2.06, 95% CI 1.23–3.45). After FDR correction across the 11 supplement categories, including probiotics, no category was statistically significant (all FDR-adjusted *p* values > 0.05 on both the OR and PR scales). This correction was applied to the exploratory comparison of supplement categories and not to the primary analysis. The negative-control analysis therefore does not clearly distinguish probiotics from all other supplements, and a partial healthy-user effect is possible. Only 14% of probiotic users also took vitamin C and 8% vitamin A/lutein, and the probiotic association was materially unchanged after mutual adjustment for any other supplement use (PR 0.57, 95% CI 0.36–0.92), for the number of other supplements (PR 0.57, 95% CI 0.36–0.91), or for multivitamin, vitamin C, omega-3, and vitamin A/lutein use simultaneously (PR 0.57, 95% CI 0.36–0.91). The E-value for the Model 2 PR was 2.9 (1.4 for the upper confidence limit), and that for Model 4 was 2.8 (1.3). For comparison, in mutually adjusted models the strongest measured correlates of DR were HbA1c ≥ 8% (PR 2.03) and diabetes duration ≥ 10 years (PR 1.99), and the strongest measured correlates of probiotic use were female sex (PR 2.21) and HbA1c ≥ 8% (PR 0.59, corresponding to 1.70 when the reference category is reversed) (Table S4). Thus, no measured covariate was associated with both probiotic use and DR as strongly as would be required to explain the point estimate, whereas an unmeasured confounder associated with both by a risk ratio of 1.4, weaker than HbA1c ≥8%, could move the confidence interval to include unity.

### 3.7. Other Retinal Diseases in the General Population

In the general cohort of 12,416–12,418 adults with gradable fundus photographs, probiotic use was not associated with AMD (adjusted OR 1.22, 95% CI 0.98–1.51), late AMD (OR 0.59, 95% CI 0.20–1.72), ERM (OR 1.15, 95% CI 0.89–1.48), or BRVO (OR 1.11, 95% CI 0.45–2.73) after adjustment for age, sex, hypertension, diabetes, smoking, and income (Table S2). Macular hole was too rare (eight cases, none among probiotic users) for analysis. These analyses were exploratory and had limited statistical power, particularly for late AMD and BRVO (four and seven exposed cases, respectively); because these outcomes were less common, ORs were reported.

## 4. Discussion

In this nationally representative study of Korean adults with diabetes, current use of a probiotic supplement was associated with a lower prevalence of DR after adjustment for age, sex, hypertension, smoking, income, diabetes duration, and HbA1c (PR 0.57, 95% CI 0.36–0.90; the PR was adopted as the main measure after the initial analysis, and the prespecified OR was 0.49, 95% CI 0.28–0.86), and the estimate changed little after further adjustment for clinical, lifestyle, and dietary factors, including fermented food intake. The estimate is based on 27 DR cases among exposed participants, and its confidence interval is compatible with associations ranging from modest to strong. Without survey weights, the association was weaker and not statistically significant (Model 2 PR 0.73, 95% CI 0.50–1.07), and the Kish effective sample size of probiotic users was 135; the weighted estimate therefore depends on the validity of the survey weights and should be interpreted with caution. In exploratory analyses, no probiotic user had VTDR (a descriptive observation in the study sample), the PR was lower among users of one year or longer than among shorter-term users, and use of other dietary supplements, considered together, was not associated with DR, although vitamin C and vitamin A/lutein supplements showed inverse associations of similar magnitude. We are not aware of previous population-based studies of probiotic supplementation in relation to DR using product-level supplement data linked to fundus photographs graded by retinal specialists. Because the design is cross-sectional, these findings indicate an association and do not show that probiotics prevent DR or slow its progression.

### 4.1. Comparison with Previous Studies

Previous human evidence linking the gut microbiome to DR has been observational and descriptive. Das et al. reported gut dysbiosis in DR, with depletion of anti-inflammatory and probiotic genera in people with DR relative to those with diabetes alone [8], and this was confirmed in Chinese and Indian cohorts, in which reduced microbial diversity and a higher Bacteroidetes to Firmicutes ratio discriminated sight-threatening DR from diabetes without retinopathy [9,10]. A systematic appraisal concluded that microbiome differences between DR and non-DR diabetes are real but smaller than those between diabetes and health, and identified probiotics and fecal microbiota transplantation as therapeutic avenues that remain untested in humans [11]. The present study addressed a related but different question, namely whether people who take probiotic supplements have less DR; they had a lower prevalence of DR, although the cross-sectional design does not allow conclusions about cause and effect.

Our findings are compatible with, but do not test, the experimental literature. In db/db mice, microbiome restructuring by intermittent fasting prevented acellular capillary formation without altering HbA1c [12], and in diabetic mouse models oral administration of genetically engineered Lactobacillus strains protected the retina [13,14,15,16]. These studies used defined strains, doses, and routes of administration that cannot be compared with the heterogeneous commercial products, of unknown strain, dose, and viability, captured by the KNHANES probiotic category; they establish biological plausibility rather than support for any particular mechanism in our participants. Randomized trials in type 2 diabetes show that probiotics modestly lower fasting glucose, insulin resistance, lipids, blood pressure, and inflammatory as well as oxidative stress markers, with small effects on HbA1c [17,18,19,31,32], and a meta-analysis of trials in diabetic kidney disease, the other major microvascular complication of diabetes, reported improvements in renal function and inflammatory markers [33]; no trial has examined retinal outcomes. The attenuation of the estimate from Model 1 to Model 2 was small, but a single HbA1c measurement does not reflect long-term glycemic exposure, and the comparison of two adjusted estimates is not a mediation analysis; our data therefore cannot determine whether the association is independent of glycemic control. Likewise, the stronger association among long-term users and the absence of VTDR among users were exploratory findings based on few events and should not be interpreted as evidence of a dose–response relationship or of an effect on disease progression.

Prior epidemiologic work on probiotics and the eye is scarce and is limited largely to dietary studies, such as case-control data relating dietary calcium sources, including dairy products, to AMD [34]. In contrast, we found no association between probiotic supplements and AMD or ERM in the Korean general population. AMD in Koreans is strongly related to age, male sex, and blood pressure [35], and to vitamin D [36], and although a gut–retina axis has been proposed for AMD [5,37], its relevance may be smaller in a population with a low prevalence of late AMD and a diet already rich in fermented foods. Because these diseases do not share the confounding structure of DR and the analyses had limited power, the null findings provide only weak evidence against a generic healthy-user explanation for the DR association.

### 4.2. Comparison with Our Previous KNHANES Studies of Diabetic Retinopathy

The present results extend a series of KNHANES analyses of DR from our group. Using seven-field mydriatic photographs in KNHANES 2008–2011, we reported an any-DR prevalence of 15.8% and a VTDR prevalence of 4.6% among Korean adults with diabetes, with diabetes duration, HbA1c, and systolic blood pressure as the principal risk factors [3]. We subsequently identified inverse associations between DR and serum 25-hydroxyvitamin D (OR 0.37 for the highest quintile) [38], between DR and sleep duration in a U-shaped fashion [39], and between DR and serum vitamin A (OR 0.32 for the highest quartile) [40]. Some of these associations were also stronger in men [38,40], but because these analyses used the same survey and similar methods, this recurrence does not constitute independent confirmation, and the sex difference observed here was not statistically robust (*p* for interaction 0.058 on the PR scale and 0.045 on the OR scale). We have also shown that dietary recall measures of nutrient intake are weaker predictors of DR than serum biomarkers [41], which underscores the value of the product-level supplement interview used here, in which the interviewer inspected each product rather than relying on recall of food categories. The 20.0% weighted DR prevalence in the present 2018–2021 cohort is higher than the 11.0% reported from single-field non-mydriatic photographs in the 2008–2009 survey [4] and the 15.8% in our earlier mydriatic study [3]; differences in photographic protocol, diabetes definition, and survey population may contribute.

### 4.3. Vision-Threatening Retinopathy

The absence of VTDR among probiotic users is of interest because VTDR, rather than mild retinopathy, determines visual outcome and treatment burden in diabetes [1,2,3], but it rests on few exposed participants and should be interpreted with caution. The design-based test gave a nominal *p* value of 0.024, but it did not account for the younger age, lower smoking prevalence, and higher income of users; the stratum-specific tests were not significant in men, in adults younger than 60 years, or in users of one year or longer (Table S1). Moreover, VTDR was absent both among users of less than one year (*n* = 122), in whom the association with any DR was weak (PR 0.76, 95% CI 0.45–1.29), and among users of one year or longer (*n* = 77); the absence of VTDR was therefore not related to the duration of use, which is an additional reason for caution. Because no adjusted estimates could be obtained for the advanced severity levels, the data do not support inferences about a gradient across disease stages or about progression. Reverse causation, whereby people with visual impairment from advanced DR are less likely to buy supplements, is also possible. The absence of VTDR among users is a descriptive observation in the present sample and does not demonstrate a protective effect. We therefore regard the VTDR finding as hypothesis-generating; larger cohorts with more exposed participants are needed before any conclusion about advanced retinopathy can be drawn.

### 4.4. Potential Mechanisms (Speculative)

The following considerations are speculative, because our data contain no information on probiotic strain, dose, or viability, or on the gut microbiome, intestinal permeability, or inflammatory markers, and no mechanism was tested in this study. In experimental models, diabetes impairs the intestinal epithelial barrier and permits translocation of microbial products that can promote retinal inflammation and capillary loss, and some probiotic strains restore barrier function, alter bile-acid metabolism, or modulate the intestinal renin–angiotensin system [6,7,12,13,14,15,16,42]. Whether the heterogeneous commercial products used by KNHANES participants have any of these effects in humans is unknown.

### 4.5. Strengths and Limitations

The strengths of this study include its nationally representative sample with complex survey weighting, product-level ascertainment of probiotic use with duration and frequency, retinal specialist grading of fundus photographs using an international severity scale, centrally measured HbA1c, design-based estimation of prevalence ratios, and a range of sensitivity, negative-control, and bias analyses. Several limitations should be acknowledged. First, the cross-sectional design precludes causal inference. Reverse causation, whereby people with DR or visual impairment change their supplement use, cannot be excluded; a similar point estimate for mild to moderate NPDR, the pattern among long-term users and the absence of an association with other retinal diseases do not rule it out. Second, some residual confounding by health-conscious behavior (the healthy-user effect) [28] is likely. Probiotic users differed from non-users in sex, smoking, income, education, physical activity, and kidney function; adjustment for these factors changed the estimate little, but vitamin C and vitamin A/lutein supplements showed inverse associations of similar magnitude, and after FDR correction the negative-control analysis did not clearly distinguish probiotics from other supplements, so a partial healthy-user effect may be present. The E-value of 2.9 exceeded the associations of all measured covariates, but the E-value of 1.4 for the upper confidence limit indicates that a confounder weaker than HbA1c ≥ 8% could make the association compatible with no difference. Diet quality and adherence to diabetes care were not measured, and fermented food intake in Model 4 was estimated from a single 24 h dietary recall, which does not capture usual intake; because recall-based intake measures are weaker predictors of DR than biomarkers [41], adjustment for dietary factors may have been incomplete. Third, the number of probiotic users with diabetes was modest (*n* = 199, with 27 cases in the primary model), so the estimates are imprecise and the subgroup, duration, and severity analyses had limited power; in addition, long-term current users are a selected group of persistent users, so the duration pattern may reflect their characteristics rather than cumulative exposure; these analyses, together with the analyses of other supplements and other retinal diseases, were exploratory and were not corrected for multiple testing except where stated, and the analysis plan was not registered; moreover, the PR was chosen as the main measure of association after the initial analysis, although the prespecified OR gave a consistent result (Model 2 OR 0.49, 95% CI 0.28–0.86). Only three probiotic users received insulin, so the results do not apply to people receiving insulin therapy. Fourth, probiotic products were classified by category only, and strain, dose, and viability were unknown. Fifth, KNHANES does not record previous treatment for DR or the type of oral glucose-lowering drug, so treated DR could not be excluded and metformin could not be examined, and 8.7% of participants with diabetes had no gradable DR assessment, which we examined with inverse probability weighting based on measured predictors of gradability. Because inverse probability weighting does not account for data missing not at random, we also performed a tipping-point analysis, in which the association was lost only if DR was considerably more common among ungradable probiotic users than among ungradable non-users (Table S5). Sixth, without survey weights the association was attenuated (Model 2 PR 0.73, 95% CI 0.50–1.07), so the weighted estimate depends on the validity of the survey weights. Seventh, the ophthalmic examination in 2021 did not include adults aged 60 years or older; the results were similar when the 2021 data were excluded. Finally, the results in Korean adults, who consume large amounts of fermented foods, may not be generalizable to other populations.

## 5. Conclusions

Probiotic supplement use was associated with a lower prevalence of DR among Korean adults with diabetes, after adjustment for sociodemographic, clinical, lifestyle, and dietary factors. In exploratory analyses, the PR was lower among long-term users, and no probiotic user had vision-threatening DR, but these findings were descriptive or exploratory, were based on few events, and do not demonstrate a protective effect. Because the study was cross-sectional and residual confounding cannot be excluded, the results are consistent with, but do not establish, a role of the gut–retina axis in DR. Prospective cohort studies with repeated assessment of probiotic use, and randomized trials with retinal endpoints, are needed to determine whether the association is causal.

## Figures and Tables

**Table 1:** Characteristics of Korean adults with diabetes according to probiotic supplement use, KNHANES 2018–2021.

Characteristic	Non-Users (*n* = 1872)	Probiotic Users (*n* = 199)	*p* Value
Age, years	60.2 ± 11.4	57.7 ± 10.3	0.005
Age ≥ 60 years	1202 (48.2)	108 (40.5)	0.10
Women	914 (40.1)	131 (58.7)	<0.001
Hypertension	1151 (57.6)	108 (51.9)	0.17
Current smoker	359 (24.6)	23 (13.2)	<0.001
Lowest household income quartile	596 (24.4)	35 (14.4)	<0.001
Body mass index, kg/m^2^	25.6 ± 3.6	25.9 ± 3.6	0.43
Physician-diagnosed diabetes	1275 (64.9)	120 (53.4)	0.013
Diabetes duration, years	6.0 ± 7.8	4.7 ± 7.7	0.065
HbA1c, %	7.2 ± 1.3	7.0 ± 1.1	0.026
Insulin use	83 (3.5)	3 (1.1)	0.002
eGFR, mL/min/1.73 m^2^	92.2 ± 17.2	97.0 ± 13.7	<0.001
College education or higher	349 (26.1)	55 (37.0)	0.027
Rural residence	483 (19.2)	38 (15.3)	0.30
Alcohol consumption ≥ 1/month	811 (49.6)	89 (51.6)	0.64
Aerobic physical activity	616 (34.5)	96 (50.2)	<0.001
Fermented food intake, g/day	144.9 ± 128.7	141.4 ± 131.2	0.77
Any fermented dairy intake	270 (14.4)	32 (15.8)	0.66
Any diabetic retinopathy	379 (21.1)	28 (11.0)	<0.001

Values are unweighted number (weighted %) or weighted mean ± SD. *p* values from design-based Wald tests accounting for the complex survey design. Hypertension: blood pressure ≥ 140/90 mmHg or antihypertensive medication. Diabetes duration was set to 0 for participants classified as having diabetes by the KDCA composite variable (fasting glucose, HbA1c, or medication use) without a physician diagnosis. eGFR, estimated glomerular filtration rate (2021 CKD-EPI creatinine equation); aerobic physical activity, meeting the national aerobic activity guideline; and fermented food intake, kimchi, fermented soybean pastes, and fermented dairy products from the 24 h dietary recall. Numbers may not sum to group totals because of missing data. KNHANES, Korea National Health and Nutrition Examination Survey.

**Table 2:** Association between probiotic supplement use and diabetic retinopathy and its severity among adults with diabetes.

Outcome	Cases/N, Users	Cases/N, Non-Users	Weighted Prevalence, Users vs. Non-Users (%)	Model 2 PR (95% CI)	Model 1 OR (95% CI)	Model 2 OR (95% CI)	*p* *
Any DR	27/197 ^a^; 27/195 ^b^	376/1850 ^a^; 372/1788 ^b^	11.0 vs. 21.1	0.57 (0.36–0.90)	0.45 (0.27–0.76)	0.49 (0.28–0.86)	0.017
Mild–moderate NPDR vs. no DR	27/197 ^a^	319/1793 ^a^	11.0 vs. 18.7	0.64 (0.40–1.02)	0.53 (0.31–0.89)	0.57 (0.33–1.00)	0.059
Severe NPDR or PDR	0/199 ^c^	58/1872 ^c^	0.0 vs. 2.9	Not estimable	—	—	0.055
PDR	0/199 ^c^	27/1872 ^c^	0.0 vs. 1.0	Not estimable	—	—	0.17
DME	0/199 ^c^	30/1872 ^c^	0.0 vs. 1.7	Not estimable	—	—	0.12
Vision-threatening DR	0/199 ^c^	72/1872 ^c^	0.0 vs. 3.9	Not estimable	—	—	0.024

PR, prevalence ratio from survey-weighted Poisson regression; OR, odds ratio from survey-weighted logistic regression. Model 1: adjusted for age, sex, hypertension, current smoking, and household income quartile (*n* = 2047). Model 2 (primary): Model 1 plus diabetes duration and HbA1c (*n* = 1983). Cases/N refer to three samples: ^a^ Model 1 complete-case sample (*n* = 2047: 197 users and 1850 non-users), in which the Model 1 OR was estimated; ^b^ Model 2 complete-case sample (*n* = 1983: 195 users and 1788 non-users), in which the Model 2 PR and OR were estimated (64 participants of the Model 1 sample lacked diabetes duration or HbA1c; for mild–moderate NPDR, the Model 2 estimates were obtained in the corresponding subset of this sample); and ^c^ full cohort (*n* = 2071: 199 users and 1872 non-users), used for severity outcomes, which were compared without covariate adjustment (24 participants lacked Model 1 covariates). Severity was classified by the worse eye. Vision-threatening DR: severe NPDR, PDR, or DME. * *p* for the Model 2 PR for any DR and mild–moderate NPDR; for severity outcomes, *p* from the design-based Rao–Scott chi-square test (not covariate-adjusted), because no event occurred among probiotic users and model-based estimates were not calculated (—). Analyses other than any DR are secondary or exploratory. DR, diabetic retinopathy; NPDR, non-proliferative DR; PDR, proliferative DR; DME, diabetic macular edema; and CI, confidence interval.

**Table 3:** Association between probiotic supplement use and any diabetic retinopathy by sex and age.

Subgroup	N	DR Cases	Users with DR/Users	Weighted DR Prevalence, Users vs. Non-Users (%)	Model 2 PR (95% CI)	*p*	Model 2 OR (95% CI)	*p*
All	1983	399	27/195	11.0 vs. 21.1	0.57 (0.36–0.90)	0.017	0.49 (0.28–0.86)	0.012
Men	993	203	7/66	6.6 vs. 20.7	0.29 (0.11–0.75)	0.011	0.22 (0.08–0.64)	0.006
Women	990	196	20/129	14.0 vs. 21.6	0.80 (0.48–1.33)	0.39	0.74 (0.39–1.41)	0.37
Age < 60 years	727	150	14/91	11.5 vs. 21.1	0.66 (0.35–1.25)	0.20	0.58 (0.27–1.26)	0.17
Age ≥ 60 years	1256	249	13/104	10.2 vs. 21.1	0.48 (0.26–0.90)	0.022	0.40 (0.19–0.84)	0.016

Domain (subpopulation) analyses of the full survey design. Model 2: adjusted for age, sex (except in sex-specific analyses), hypertension, current smoking, household income quartile, diabetes duration, and HbA1c. PR, prevalence ratio (survey-weighted Poisson regression); OR, odds ratio (survey-weighted logistic regression). *p* for interaction (design-based Wald test of the product term, PR and OR scale): sex, 0.058 and 0.045; and age group, 0.67 and 0.64. Subgroup analyses are exploratory, and *p* values are not adjusted for multiple testing. No probiotic user in any subgroup had vision-threatening DR. DR, diabetic retinopathy; CI, confidence interval.

**Table 4:** Sensitivity analyses of the association between probiotic supplement use and any diabetic retinopathy.

Analysis	N	Exposed (DR Cases)	OR (95% CI)	*p*	PR (95% CI)	*p*
Primary analysis: Model 2, current probiotic use vs. no probiotic use	1983	195 (27)	0.49 (0.28–0.86)	0.012	0.57 (0.36–0.90)	0.017
Model 1	2047	197 (27)	0.45 (0.27–0.76)	0.003	0.51 (0.32–0.81)	0.004
Model 3: Model 2 plus BMI, insulin use, eGFR, education, residence, alcohol consumption, and physical activity	1855	189 (27)	0.51 (0.28–0.91)	0.023	0.59 (0.37–0.95)	0.030
Model 2 in the Model 3 sample	1855	189 (27)	0.51 (0.29–0.91)	0.022	0.59 (0.37–0.95)	0.029
Model 4: Model 3 plus fermented food and any fermented dairy intake	1853	189 (27)	0.51 (0.28–0.91)	0.023	0.59 (0.37–0.95)	0.030
Model 3 with inverse probability weighting for gradability and missing covariates	1855	189 (27)	0.51 (0.29–0.91)	0.023	0.60 (0.37–0.95)	0.030
Probiotic use < 1 year vs. non-users	1983	119 (20)	0.70 (0.36–1.34)	0.28	0.76 (0.45–1.29)	0.30
Probiotic use ≥ 1 year vs. non-users	1983	76 (7)	0.21 (0.09–0.50)	<0.001	0.28 (0.13–0.62)	0.002
*p* for trend across duration categories				0.001		0.003
Probiotic use ≥ 1 year vs. <1 year (contrast within the three-category model)	1983	76 (7)	0.30 (0.11–0.83)	0.021	0.37 (0.15–0.91)	0.031
Probiotic users vs. users of no dietary supplement	1163	195 (27)	0.50 (0.28–0.89)	0.019	0.58 (0.36–0.94)	0.026
Excluding 61 participants not meeting the composite diabetes definition (Model 1)	1986	195 (27)	0.44 (0.26–0.74)	0.002	0.50 (0.32–0.80)	0.003
Excluding participants with previous eye surgery	1440	154 (22)	0.53 (0.29–0.98)	0.043	0.61 (0.36–1.01)	0.055
Excluding insulin users	1897	192 (26)	0.50 (0.28–0.88)	0.017	0.57 (0.36–0.92)	0.023
Model 3 without the insulin-use term	1855	189 (27)	0.50 (0.28–0.89)	0.019	0.58 (0.36–0.93)	0.024
Model 3 without the insulin-use term, excluding insulin users	1779	186 (26)	0.50 (0.28–0.91)	0.023	0.58 (0.36–0.95)	0.029
Adults aged ≥ 40 years	1967	193 (27)	0.48 (0.27–0.85)	0.011	0.57 (0.36–0.90)	0.016
Additionally adjusted for survey year	1983	195 (27)	0.48 (0.27–0.84)	0.011	0.56 (0.35–0.90)	0.016
Additionally adjusted for oral glucose-lowering drug and insulin use	1983	195 (27)	0.53 (0.30–0.92)	0.024	0.60 (0.38–0.96)	0.033
Hypertension defined by antihypertensive medication use alone	1987	195 (27)	0.49 (0.28–0.86)	0.012	0.57 (0.36–0.90)	0.017
Physician-diagnosed diabetes only	1373	119 (19)	0.40 (0.22–0.76)	0.005	0.50 (0.30–0.83)	0.008
Survey years 2018–2020 only	1802	170 (23)	0.46 (0.25–0.84)	0.011	0.54 (0.32–0.89)	0.016
Survey weights truncated at the 99th percentile	1983	195 (27)	0.49 (0.28–0.86)	0.012	0.57 (0.36–0.90)	0.017
Survey weights truncated at the 95th percentile	1983	195 (27)	0.50 (0.29–0.88)	0.015	0.58 (0.37–0.92)	0.021
Without survey weights, Model 1	2047	197 (27)	0.62 (0.40–0.96)	0.034	0.67 (0.46–0.98)	0.039
Without survey weights, Model 2	1983	195 (27)	0.67 (0.42–1.07)	0.096	0.73 (0.50–1.07)	0.11

All analyses use Model 2 unless stated otherwise. Model 1: adjusted for age, sex, hypertension, current smoking, and household income quartile. Model 2: Model 1 plus diabetes duration and HbA1c. OR, odds ratio (survey-weighted logistic regression); PR, prevalence ratio (survey-weighted Poisson regression). Inverse probability weights were multiplied by the survey weights. Analyses without survey weights retained strata and clusters. Duration and sensitivity analyses are exploratory. E-values for the Model 2 PR (point estimate; upper confidence limit): 2.9 (1.4); Model 4: 2.8 (1.3). BMI, body mass index; eGFR, estimated glomerular filtration rate; DR, diabetic retinopathy; and CI, confidence interval.

**Table 5:** Negative-control analyses: association of other dietary supplement categories with diabetic retinopathy, and probiotic estimates after mutual adjustment.

Exposure	Users, n	DR Cases Among Users	Model 2 PR (95% CI)	Model 2 OR (95% CI)	*p* for OR (FDR-Adjusted *p*)
Probiotics (primary exposure)	199	28	0.57 (0.36–0.90)	0.49 (0.28–0.86)	0.012 (0.10)
Any non-probiotic supplement	1003	188	0.92 (0.75–1.14)	0.88 (0.66–1.18)	0.40
Multivitamin-mineral	411	75	0.94 (0.72–1.22)	0.90 (0.63–1.28)	0.55 (0.75)
Omega-3 fatty acids	339	72	1.10 (0.84–1.44)	1.16 (0.80–1.69)	0.44 (0.69)
Other supplements	336	67	1.06 (0.81–1.38)	1.09 (0.75–1.59)	0.64 (0.79)
Vitamin C	199	29	0.62 (0.39–1.00)	0.55 (0.31–0.97)	0.038 (0.10)
Calcium	111	22	0.96 (0.62–1.49)	0.94 (0.51–1.75)	0.86 (0.86)
Red ginseng	106	21	0.95 (0.63–1.43)	0.91 (0.51–1.65)	0.76 (0.84)
Vitamin A/lutein	104	16	0.58 (0.35–0.98)	0.50 (0.27–0.93)	0.029 (0.10)
Vitamin D	55	6	0.46 (0.17–1.20)	0.38 (0.13–1.16)	0.088 (0.19)
Other vitamins and minerals	51	10	0.81 (0.44–1.48)	0.70 (0.31–1.57)	0.39 (0.69)
Propolis	34	12	2.06 (1.23–3.45)	3.01 (1.12–8.05)	0.029 (0.10)
Probiotics, additionally adjusted for any other supplement use	199	28	0.57 (0.36–0.92)	0.49 (0.28–0.87)	0.015
Probiotics, additionally adjusted for number of other supplements	199	28	0.57 (0.36–0.91)	0.49 (0.28–0.87)	0.014
Probiotics, additionally adjusted for multivitamin, vitamin C, omega-3, and vitamin A/lutein use	199	28	0.57 (0.36–0.91)	0.49 (0.28–0.86)	0.013

Adults with diabetes and gradable fundus photographs (*n* = 2071; Model 2 *n* = 1983). Each supplement category was entered as the exposure in place of probiotics. Model 2: adjusted for age, sex, hypertension, current smoking, household income quartile, diabetes duration, and HbA1c. PR, prevalence ratio (survey-weighted Poisson regression); OR, odds ratio (survey-weighted logistic regression). Users and DR cases among users refer to the full cohort; the Model 2 sample included 195 probiotic users with 27 cases. *p* values are for the OR; values in parentheses are Benjamini–Hochberg false discovery rate (FDR)-adjusted *p* values across the 11 individual supplement categories, including probiotics (on the PR scale, the smallest FDR-adjusted *p* was 0.068 for propolis and 0.094 for probiotics). These analyses are exploratory. The FDR correction applies to this exploratory comparison only and not to the primary analysis. Iron (1 user) was not analyzed. DR, diabetic retinopathy; CI, confidence interval.

## Data Availability

The KNHANES data analyzed in this study are publicly available from the Korea Disease Control and Prevention Agency (https://knhanes.kdca.go.kr, accessed on 22 October 2026). The R code used for the analyses is available from the corresponding author on reasonable request.

## Supplementary Materials

The following supporting information can be downloaded at: https://www.mdpi.com/article/10.3390/nu18213501/s1, Figure S1: Directed acyclic graph summarizing the assumptions underlying covariate selection, constructed after the initial analysis; Table S1: Vision-threatening diabetic retinopathy among probiotic users and non-users by subgroup, survey year, and duration of use; Table S2: Association between probiotic supplement use and other retinal diseases in the general adult population (exploratory outcomes); Table S3: Characteristics of participants with diabetes with and without a gradable diabetic retinopathy assessment; Table S4: Associations of measured covariates with diabetic retinopathy and with probiotic supplement use (E-value benchmarking); Table S5: Tipping-point analysis for participants with diabetes without a gradable diabetic retinopathy assessment.

## Abbreviations

The following abbreviations are used in this manuscript:

**Abbreviation**	**Definition**
ACE2	Angiotensin-converting enzyme 2
AMD	Age-related macular degeneration
BMI	Body mass index
BRVO	Branch retinal vein occlusion
CI	Confidence interval
CKD-EPI	Chronic Kidney Disease Epidemiology Collaboration
DAG	Directed acyclic graph
DME	Diabetic macular edema
DR	Diabetic retinopathy
eGFR	Estimated glomerular filtration rate
ERM	Epiretinal membrane
FDR	False discovery rate
HbA1c	Glycated hemoglobin
IRB	Institutional Review Board
KDCA	Korea Disease Control and Prevention Agency
KNHANES	Korea National Health and Nutrition Examination Survey
NPDR	Non-proliferative diabetic retinopathy
OR	Odds ratio
PDR	Proliferative diabetic retinopathy
PR	Prevalence ratio
PSU	Primary sampling unit
SD	Standard deviation
TGR5	Takeda G protein-coupled receptor 5
VTDR	Vision-threatening diabetic retinopathy
