# Supplementary material for: Probiotic Supplement Use and Diabetic Retinopathy in Korean Adults with Diabetes: A Nationwide Population-Based Study
Source: Nutrients. 2026 Oct 25;18(21):3501. doi: 10.3390/nu18213501 (PMC13765001; doi:10.3390/nu18213501)
Supplement: Supplementary file 1 [file nutrients-18-03501-s001.zip › nutrients-4637357_Supplementary_R2.pdf]

Supplementary Materials

Probiotic Supplement Use and Diabetic Retinopathy in Korean Adults With Diabetes: A Nationwide Population-Based Study

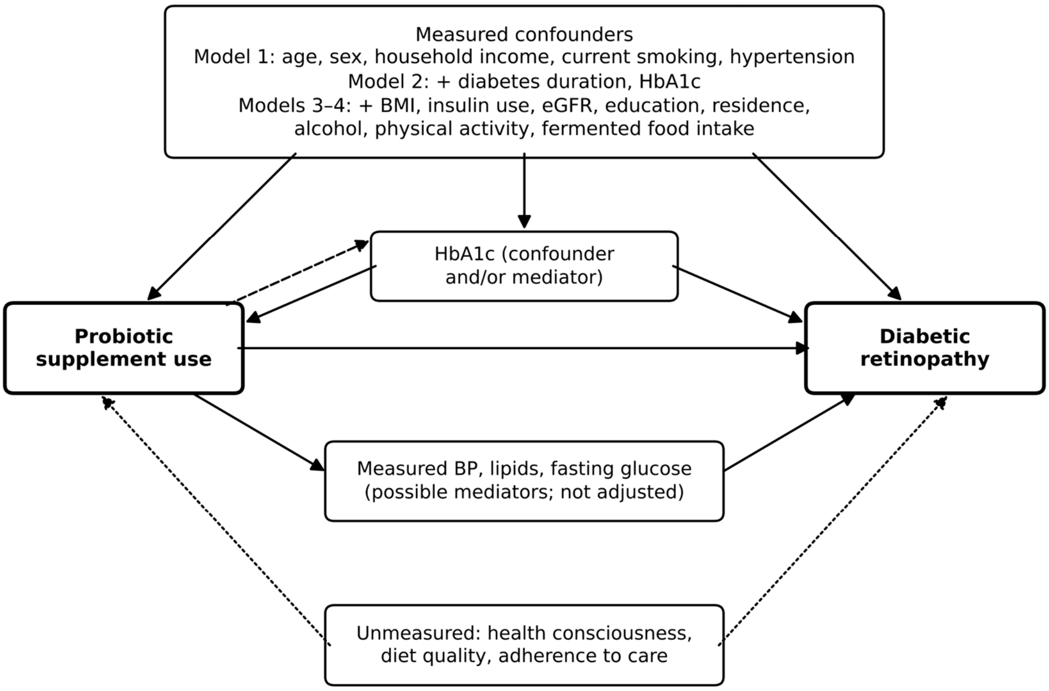

Solid arrows, assumed causal paths; dashed arrow, possible effect of probiotics on glycemia; dotted arrows, unmeasured confounding.
BP, blood pressure; BMI, body mass index; eGFR, estimated glomerular filtration rate; HbA1c, glycated hemoglobin.

Figure S1. Directed acyclic graph summarizing the assumptions underlying covariate selection (constructed after the initial analysis). The covariates of Models 1 and 2, including the primary model (Model 2), were selected before the analysis on the basis of established risk factors for diabetic retinopathy and determinants of supplement use. After the initial analysis, these assumptions were summarized in this graph, which was then used to select the additional covariates of Models 3 and 4; the graph therefore did not determine the primary model. The graph summarizes the assumed relations between probiotic supplement use, diabetic retinopathy and other variables. Age, sex, household income, current smoking and hypertension (KDCA definition: measured blood pressure ≥140/90 mmHg or antihypertensive medication; medication use alone in a sensitivity analysis) were regarded as confounders (Model 1). Diabetes duration and HbA1c were added in Model 2; HbA1c may act as a confounder or as a mediator, and a single measurement cannot separate these roles. Blood pressure, lipid and fasting glucose levels measured at the survey were regarded as possible mediators, because probiotics modify them in randomized trials, and were not adjusted for. Models 3 and 4 added clinical, socioeconomic, lifestyle and dietary factors. Health consciousness, diet quality and adherence to diabetes care were not measured.

Table S1. Vision-threatening diabetic retinopathy among probiotic users and non-users by subgroup, survey year and duration of use.

Stratum	VTDR cases / probiotic users	VTDR cases / non-users (unweighted %)	Weighted VTDR prevalence in non-users (%)	Expected cases among users *	Fisher exact P †
All	0 / 199	72 / 1,872 (3.8)	3.9	7.7	0.0009
Men	0 / 68	34 / 958 (3.5)	3.3	2.4	0.16
Women	0 / 131	38 / 914 (4.2)	4.7	5.4	0.010
Age <60 years	0 / 91	27 / 670 (4.0)	4.2	3.7	0.063
Age ≥60 years	0 / 108	45 / 1,202 (3.7)	3.5	4.0	0.046
Survey year 2018	0 / 25	33 / 567 (5.8)	5.6	1.5	0.39
Survey year 2019	0 / 58	16 / 582 (2.7)	2.4	1.6	0.38
Survey year 2020	0 / 91	15 / 565 (2.7)	2.8	2.4	0.25
Survey year 2021	0 / 25	8 / 158 (5.1)	4.6	1.3	0.60
Probiotic use <1 year vs non-users	0 / 122	72 / 1,872 (3.8)	3.9	4.7	0.020
Probiotic use ≥1 year vs non-users	0 / 77	72 / 1,872 (3.8)	3.9	3.0	0.11

VTDR, vision-threatening diabetic retinopathy (severe non-proliferative DR, proliferative DR or diabetic macular edema in either eye). *Number of VTDR cases expected among probiotic users if their proportion with VTDR equaled the unweighted proportion among non-users in the same stratum (number of probiotic users × VTDR cases / non-users; unweighted proportion shown in parentheses). The weighted prevalence among non-users is shown for description and was not used in this calculation. †Unweighted Fisher exact tests, reported for descriptive purposes; they do not account for the complex survey design or for differences in covariates between users and non-users. With the design-based Rao–Scott chi-square test, the nominal P = 0.024 for all participants. The exact 95% CI for the proportion of probiotic users with VTDR was 0–1.8%. The analyses are exploratory and are not adjusted for multiple testing.

Table S2. Association between probiotic supplement use and other retinal diseases in the general adult population (exploratory outcomes).

Outcome	N	Cases	Users with disease / users	Weighted prevalence, users vs non-users (%)	Crude OR (95% CI)	Adjusted OR (95% CI)	P
Any AMD	12,416	1,757	186 / 1,678	9.7 vs 12.1	0.78 (0.64–0.96)	1.22 (0.98–1.51)	0.077
Late AMD	12,416	108	4 / 1,678	0.2 vs 0.8	0.26 (0.09–0.73)	0.59 (0.20–1.72)	0.34
Epiretinal membrane	12,418	1,029	103 / 1,678	5.0 vs 6.7	0.74 (0.58–0.94)	1.15 (0.89–1.48)	0.28
Branch retinal vein occlusion	12,418	70	7 / 1,678	0.5 vs 0.6	0.89 (0.40–1.98)	1.11 (0.45–2.73)	0.82
Macular hole	12,418	8	0 / 1,678	0.0 vs 0.1	Not estimable	Not estimable	—

Adults aged ≥19 years with gradable fundus photographs and dietary supplement data. Adjusted for age, sex, hypertension, diabetes, current smoking and household income quartile, with survey weights. These analyses are exploratory and have limited statistical power for late AMD and branch retinal vein occlusion; because these outcomes were less common, ORs are reported. AMD, age-related macular degeneration; OR, odds ratio; CI, confidence interval.

Table S3. Characteristics of participants with diabetes with and without a gradable diabetic retinopathy assessment.

Characteristic	No gradable assessment (n = 192)	Gradable assessment (n = 2,010)	P
Age, years	70.8 ± 9.7	63.3 ± 11.1	<0.001
Women	89 (46.4)	1,007 (50.1)	0.36
HbA1c, %	7.4 ± 1.5	7.2 ± 1.3	0.029
Diabetes duration, years	10.6 ± 11.0	6.7 ± 8.5	<0.001
Hypertension	132 (69.5)	1,222 (60.9)	0.026
Insulin use	17 (8.9)	86 (4.3)	0.007
Current smoker	28 (15.0)	370 (18.6)	0.27
Household income quartile (1–4)	2.0 ± 1.1	2.3 ± 1.1	<0.001
Rural residence	39 (20.3)	505 (25.1)	0.16
Body mass index, kg/m^2	25.0 ± 3.6	25.5 ± 3.6	0.077
eGFR, mL/min/1.73 m^2	79.3 ± 21.0	89.9 ± 17.2	<0.001
Probiotic supplement use	7 (3.6)	197 (9.8)	0.007

Participants with diabetes meeting the composite definition and with dietary supplement data, KNHANES 2018–2021. Values are unweighted number (%) or mean ± SD; percentages are calculated among participants with non-missing data. P values from the Welch t-test or the chi-square test. eGFR, estimated glomerular filtration rate (2021 CKD-EPI creatinine equation).

Table S4. Associations of measured covariates with diabetic retinopathy and with probiotic supplement use (E-value benchmarking).

Covariate	PR (95% CI) for diabetic retinopathy	PR (95% CI) for probiotic use
Female sex	1.18 (0.95–1.47)	2.21 (1.53–3.17)
Age, per year	1.00 (0.99–1.01)	0.98 (0.96–0.99)
Hypertension	1.05 (0.84–1.32)	0.98 (0.72–1.34)
Current smoking	1.29 (0.97–1.72)	0.63 (0.35–1.11)
Household income, per quartile	1.07 (0.96–1.18)	1.26 (1.07–1.48)
Diabetes duration ≥10 years	1.99 (1.59–2.49)	0.95 (0.64–1.42)
HbA1c ≥8%	2.03 (1.64–2.51)	0.59 (0.36–0.95)
Probiotic supplement use	0.56 (0.35–0.90)	—

Prevalence ratios (PRs) from mutually adjusted survey-weighted Poisson regression models among adults with diabetes (n = 1,983), with diabetes duration and HbA1c categorized (≥10 years and ≥8%); the probiotic PR (0.56) therefore differs slightly from the Model 2 estimate (0.57). The PRs for probiotic use approximate the covariate–exposure associations used in E-value benchmarking. The E-value for the primary Model 2 PR of 0.57 (95% CI 0.36–0.90) was 2.9 for the point estimate and 1.4 for the upper confidence limit: an unmeasured confounder would need to be associated with both probiotic use and diabetic retinopathy by a risk ratio of at least 2.9 each, beyond the measured covariates, to fully explain the point estimate. No measured covariate reached this strength for both associations; HbA1c ≥8% was the strongest (PR 2.03 for retinopathy, and 1.70 for probiotic use when the reference category is reversed). CI, confidence interval.

Table S5. Tipping-point analysis for participants with diabetes without a gradable diabetic retinopathy assessment: Model 2 prevalence ratio (95% CI) for any diabetic retinopathy according to the number of ungradable probiotic users and the prevalence among ungradable non-users assigned diabetic retinopathy.

DR prevalence among ungradable non-users	0/7 (0%)	1/7 (14%)	2/7 (29%)	3/7 (43%)	4/7 (57%)	5/7 (71%)	6/7 (86%)	7/7 (100%)
10%	0.56 (0.35–0.89)	0.59 (0.38–0.93)	0.63 (0.40–0.98)	0.66 (0.43–1.01)*	0.69 (0.45–1.05)*	0.73 (0.48–1.10)*	0.76 (0.51–1.14)*	0.80 (0.54–1.18)*
20%	0.54 (0.34–0.87)	0.58 (0.37–0.91)	0.62 (0.40–0.96)	0.64 (0.42–0.99)	0.68 (0.44–1.03)*	0.71 (0.47–1.07)*	0.74 (0.50–1.11)*	0.78 (0.53–1.15)*
30%	0.53 (0.33–0.85)	0.56 (0.36–0.89)	0.59 (0.38–0.92)	0.62 (0.41–0.96)	0.66 (0.43–1.00)*	0.69 (0.46–1.04)*	0.73 (0.49–1.08)*	0.76 (0.51–1.12)*
40%	0.52 (0.33–0.82)	0.55 (0.35–0.86)	0.58 (0.38–0.91)	0.61 (0.40–0.94)	0.64 (0.42–0.98)	0.67 (0.45–1.01)*	0.70 (0.47–1.05)*	0.74 (0.50–1.09)*
50%	0.50 (0.32–0.80)	0.54 (0.34–0.84)	0.57 (0.36–0.88)	0.60 (0.39–0.92)	0.63 (0.41–0.95)	0.66 (0.44–0.99)	0.69 (0.46–1.02)*	0.72 (0.49–1.05)*
60%	0.50 (0.31–0.79)	0.52 (0.33–0.82)	0.55 (0.36–0.86)	0.58 (0.38–0.90)	0.61 (0.40–0.93)	0.64 (0.43–0.96)	0.67 (0.45–1.00)	0.70 (0.48–1.03)*

Of the 192 participants who met the composite diabetes definition, had dietary supplement data and had no gradable DR assessment, 180 (7 probiotic users and 173 non-users) had complete Model 2 covariates and were added to the Model 2 sample (n = 1,983), giving a combined sample of 2,163. Columns show the number (%) of the 7 ungradable probiotic users to whom DR was assigned; rows show the DR prevalence assigned to the ungradable non-users. For each scenario, DR status was assigned at random 50 times (to the stated number of probiotic users and with the stated probability among non-users), Model 2 was estimated with survey-weighted Poisson regression as a domain analysis of the full survey design, and the results were combined with Rubin's rules. The primary Model 2 estimate in participants with a gradable assessment was 0.57 (95% CI 0.36–0.90). *95% CI includes unity (the upper limit for 30% and 4/7 was 1.001, and that for 60% and 6/7 was 0.999, before rounding). DR, diabetic retinopathy; CI, confidence interval.
